# Melatonin inhibits NaIO_3_-induced ARPE-19 cell apoptosis via suppression of HIF-1α/BNIP3-LC3B/mitophagy signaling

**DOI:** 10.1186/s13578-022-00879-3

**Published:** 2022-08-19

**Authors:** Kai Wang, Yong-Syuan Chen, Hsiang-Wen Chien, Hui-Ling Chiou, Shun-Fa Yang, Yi-Hsien Hsieh

**Affiliations:** 1 Department of Ophthalmology, Cathay General Hospital, Taipei, Taiwan; 2 Departments of Ophthalmology, Sijhih Cathay General Hospital, New Taipei City, Taiwan; 3 School of Medicine, College of Medicine, Fu Jen Catholic University, New Taipei City, Taiwan; 4 Institute of Medicine, Chung Shan Medical University, Taichung, Taiwan; 5 School of Medicine, National Tsing Hua University, Hsinchu, Taiwan; 6 Department of Medical Laboratory and Biotechnology, Chung Shan Medical University, Taichung, Taiwan; 7 Department of Medical Research, Chung Shan Medical University Hospital, Taichung, Taiwan

**Keywords:** Melatonin, Retinal pigment epithelial cells, BNIP3, HIF-1α, LC3B, Mitophagy, Age-related macular degeneration

## Abstract

**Background:**

Age-related macular degeneration (AMD) leads to gradual central vision loss and eventual irreversible blindness. Melatonin, an endogenous hormone, exhibits anti-inflammatory and antitumor effects; however, the role it plays in AMD remains unclear. Herein, we investigated the anti-AMD molecular mechanism of melatonin after sodium iodate (NaIO3) treatment of ARPE-19 cells in vitro and in animal models with the goal of improving the therapeutic effect.

**Results:**

The in vitro results showed that melatonin protected against NaIO_3_-induced cell viability decline, mitochondrial dysfunction and apoptosis in ARPE-19 cells, and melatonin also alleviated NaIO_3_-induced reactive oxygen species (ROS) production, mitochondrial dysfunction and mitophagy activation. Melatonin reduced NaIO_3_-induced mitophagy activation through HIF-1α-targeted BNIP3/LC3B transcription, whereas ROS inhibition realized with N-acetylcysteine (NAC, a ROS inhibitor) combined with melatonin reduced the effect of NaIO_3_ on mitophagy. An animal model of AMD was established to confirm the in vitro data. Mouse tail vein injection of NaIO_3_ and melatonin was associated with enhanced repair of retinal layers within 7 days, as observed by optical coherence tomography (OCT) and hematoxylin and eosin (H&E) staining. A reduction in BNIP3 and HIF-1α levels, as determined by immunohistochemistry (IHC) assay, was also observed.

**Conclusions:**

These results indicate that melatonin attenuated NaIO_3_-induced mitophagy of ARPE-19 cells via reduction in ROS-mediated HIF-1α targeted BNIP3/LC3B signaling in vitro and in vivo. Melatonin may be a potential therapeutic drug in the treatment of AMD.

**Supplementary Information:**

The online version contains supplementary material available at 10.1186/s13578-022-00879-3.

## Introduction

Age-related macular degeneration (AMD) is the major cause of vision damage and blindness in elderly people in developed countries [1]. The most important risk factors associated with AMD are age, oxidative stress, inflammation, and genetic factors [2]. Additionally, retinal pigment epithelium (RPE) cells, also known as monolayer pigmented cells, play an important role in providing nutrients to the retina and overall health to photoreceptors of the eyes [3]. Abnormalities in physiological function and reactive oxygen species (ROS) production in RPE cells contribute to vision damage and subsequently to AMD; however, the pathophysiology of AMD remains unclear [4, 5].

Sodium iodate (NaIO_3_) is a strong oxidizing agent that has been extensively used in preclinical experimental models of RPE dystrophy in vivo and in vitro. NaIO_3_ animal models have been used to investigate the mechanism associated with AMD pathogenesis because NaIO_3_ effectively produces large quantities of ROS in RPE cells in various animal species, including mice, sheep, and rabbits [6, 7]. Previous reports showed that NaIO_3_-induced ROS production affected the function of photoreceptors and the choriocapillaris, contributing to RPE cell damage [8]. Moreover, a previous study showed that several natural remedies (e.g., glycyrrhizin) relieved RPE cell damage induced by NaIO_3_ treatment. Glycyrrhizin attenuated NaIO_3_‐induced RPE and retinal injury through AKT and Nrf2/HO-1 signaling in vitro and in vivo [9]. In addition, αB crystallin, a biomarker of advanced AMD [10], effectively protected against NaIO_3_‐induced retinal degeneration [11]. Therefore, NaIO_3_ has been widely used to study the molecular mechanism of RPE cell death in AMD.

Melatonin is an important hormone secreted by the pineal gland and regulates physiological circadian rhythms in humans [12]. Melatonin has antioxidant [13], anti-inflammatory [14], anti‐proliferative [15], anti‐metastatic [16] and pro-apoptotic [17] effects during tumorigenesis and in several diseases. Melatonin that is produced in the retina [18], and may play a key role in retinal homeostasis. n clinical research, 6 months of treatment with melatonin (3 mg/day), a patient's AMD was reversed due to retinal protection and macular regeneration with no significant side effects [19]. Previous research in our laboratory demonstrated that melatonin repressed EGF-induced cathepsin S expression in a cell model of proliferative vitreoretinopathy (PVR), [20], which is considered to be a predecessor of AMD. Melatonin has been hypothesized to rebuild telomeres via activation of telomerase in the retina of patients with AMD [21], suggesting an important role for melatonin in AMD treatment.

ROS, including free radicals, are primary risk factors for AMD. Increased ROS levels in RPE cells strongly promoted oxidative damage in mitochondrial DNA (mtDNA) [22], causing extensive mtDNA damage that induced eye disease in animal models.[23]. Furthermore, ROS enhanced the expression of HIF-1α by mediating increased binding activity of NF-κB at the HIF-1α-promoter. Other factors, including VEGF [24], ANGPT [25] and MMPs [26, 27] directly contributed to AMD and have been associated with HIF targets. However, the relationships among ROS, HIF-1α, and AMD remains unclear. In this study, the ARPE-19 human RPE cell line was co-treated with NaIO_3_ and melatonin to investigate the in vitro antioxidant and antiapoptotic effects of melatonin. In addition, a NaIO_3_-induced AMD-like animal model was established to analyze the mechanism associated with RPE and photoreceptor cell death in vivo.

## Results

### Melatonin decreases NaIO_3_-induced ARPE-19 cell death.

**Fig. 1:**
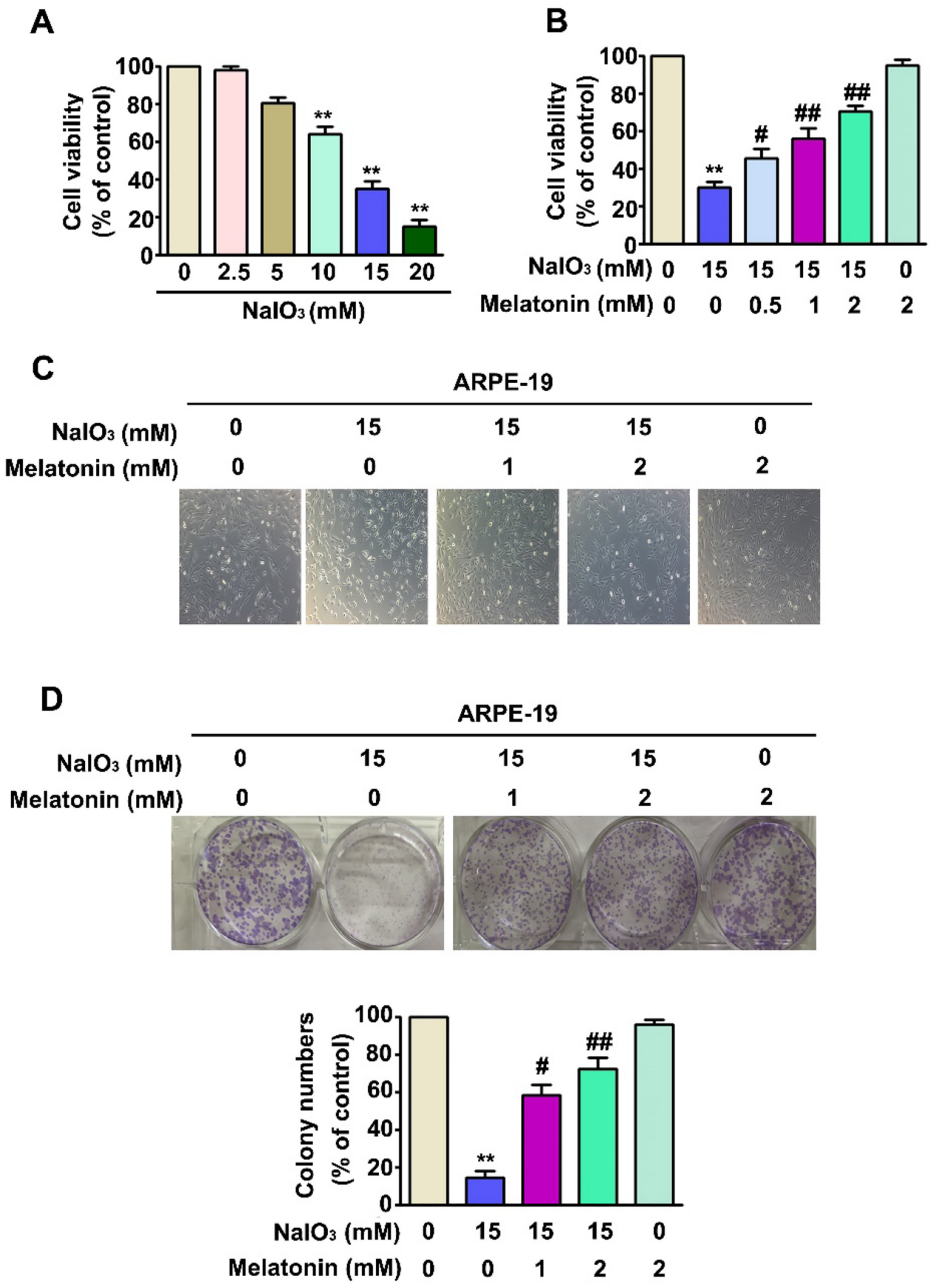
The effects of NaIO_3_ alone or in combination with melatonin on the proliferation of ARPE-19 cells. **A** ARPE-19 cells were treated with NaIO_3_ (0, 2.5, 5, 10, 15, and 20 mM) for 24 h; cell viability was measured by MTT assay. **B** ARPE-19 cells were cotreated with NaIO_3_ (15 mM) and melatonin at a series of concentrations (0, 0.5, 1, and 2 mM) for 24 h; cell viability was evaluated by MTT assay. **C** The morphology of ARPE-19 cells cotreated with NaIO_3_ (15 mM) and melatonin at a series of concentrations (0, 1 and 2 mM) for 24 h. **D** The cell proliferation rate was determined from the results of a colony formation assay. All of the data are presented as the mean ± SEM of three independent experiments. **, P < 0.01 compared with the control; #, P < 0.05, ##, P < 0.01 compared with the NaIO_3_ treatment group

For effectively treating AMD-like cells in culture with NaIO_3_ [28, 29], an MTT assay was performed to determine optimal NaIO_3_ concentrations (2.5, 5, 10, 15 and 20 mM) and eliminate those that might cause cytotoxicity in ARPE-19 cells. The results showed that NaIO_3_ treatment of 15 and 20 mM led to significant toxicity in ARPE-19 cells (Fig. 1A). In addition, our previous study showed that melatonin at lower concentrations (0 ~ 2 mM) exerted a protective effect on ARPE-19 cells by attenuating the abnormal progression without inducing cytotoxicity. To explore the protective effect of melatonin against NaIO_3_-induced cell injury, cells were pretreated with low concentrations of NaIO_3_ (0.5, 1 and 2 mM) for 2 h and then treated with a higher NaIO_3_ concentration (15 mM) for an additional 22 h. The results of this combinatory treatment showed that melatonin strongly inhibited NaIO_3_-induced cell death (Fig. 1B) and cell morphology changes (Fig. 1C). A colony formation assay was performed to confirm the protective effect of the combination treatment on ARPE-19 cells. Cotreatment with melatonin increased the proliferation of ARPE-19 cells (Fig. 1D). These results indicated that melatonin significantly reduced the NaIO_3_-induced ARPE-19 cell death rate.

### Melatonin inhibits NaIO_3_-induced apoptosis in ARPE-19 cells

**Fig. 2:**
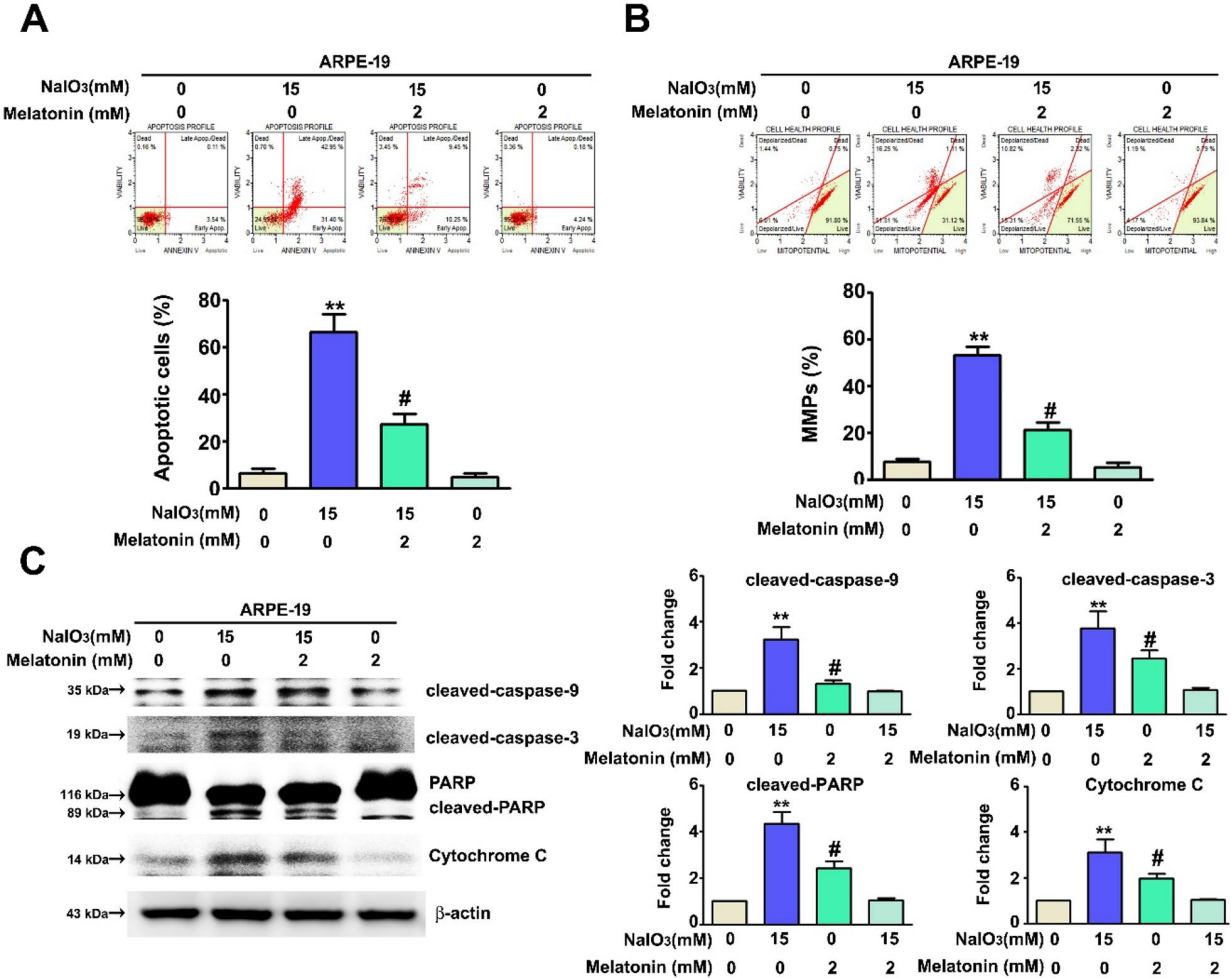
Melatonin reduced NaIO_3_-induced apoptosis in ARPE-19 cells. After combination treatment with NaIO_3_ and melatonin, an Annexin-V/PI assay and MitoPotential kit were used to detect **A** cell apoptosis. The Y-axis presents the cell viability (%) of normal healthy cells (LL), early and apoptotic cells (LR/UR) and necrotic cells (UL), and **B** the mitochondrial membrane potential, (upper left): percentage of dead cells with intact mitochondrial membrane; (lower left): percentage of live cells with depolarized mitochondrial membranes. **C** Cell lysates were analyzed and cleaved caspase-9, cleaved caspase-3, cleaved PARP and cytochrome C was detected by using western blotting (β-actin was used as the internal control). All of the data are presented as the mean ± SEM of three independent experiments. **, P < 0.01 compared with control group, and #, P < 0.05 compared with the NaIO_3_ treatment group

An Annexin-V/PI assay and measurement of mitochondrial membrane potential were performed to investigate the mechanism through which NaIO_3_ treatment induced cell death. Treatment with melatonin increased the cell survival rate via the inhibition of NaIO_3_-induced apoptosis (Fig. 2A). Mitochondrial depolarization was also observed. Melatonin treatment reduced the proportion of ARPE-19 cells with depolarized mitochondria (Fig. 2B). The levels of apoptosis-associated proteins were also examined. Following administration of cotreatment, melatonin reduced the protein levels of cleaved-caspase-9, cleaved-caspase-3, cleaved-PARP, and total cytochrome c, which had been activated by NaIO_3_ treatment (Fig. 2C). However, we found increased expression of mitochondrial cytochrome c in ARPE-19 cells treated with NaIO_3_ combined with melatonin (Additiional file 1: Fig S1). Taken together, these data demonstrated that melatonin inhibited NaIO_3_-induced cell apoptosis via inactivation of apoptosis-associated proteins in ARPE-19 cells.

### Melatonin reduces NaIO_3_-induced cell apoptosis through inhibition of HIF-1α expression in ARPE-19 cells

**Fig. 3:**
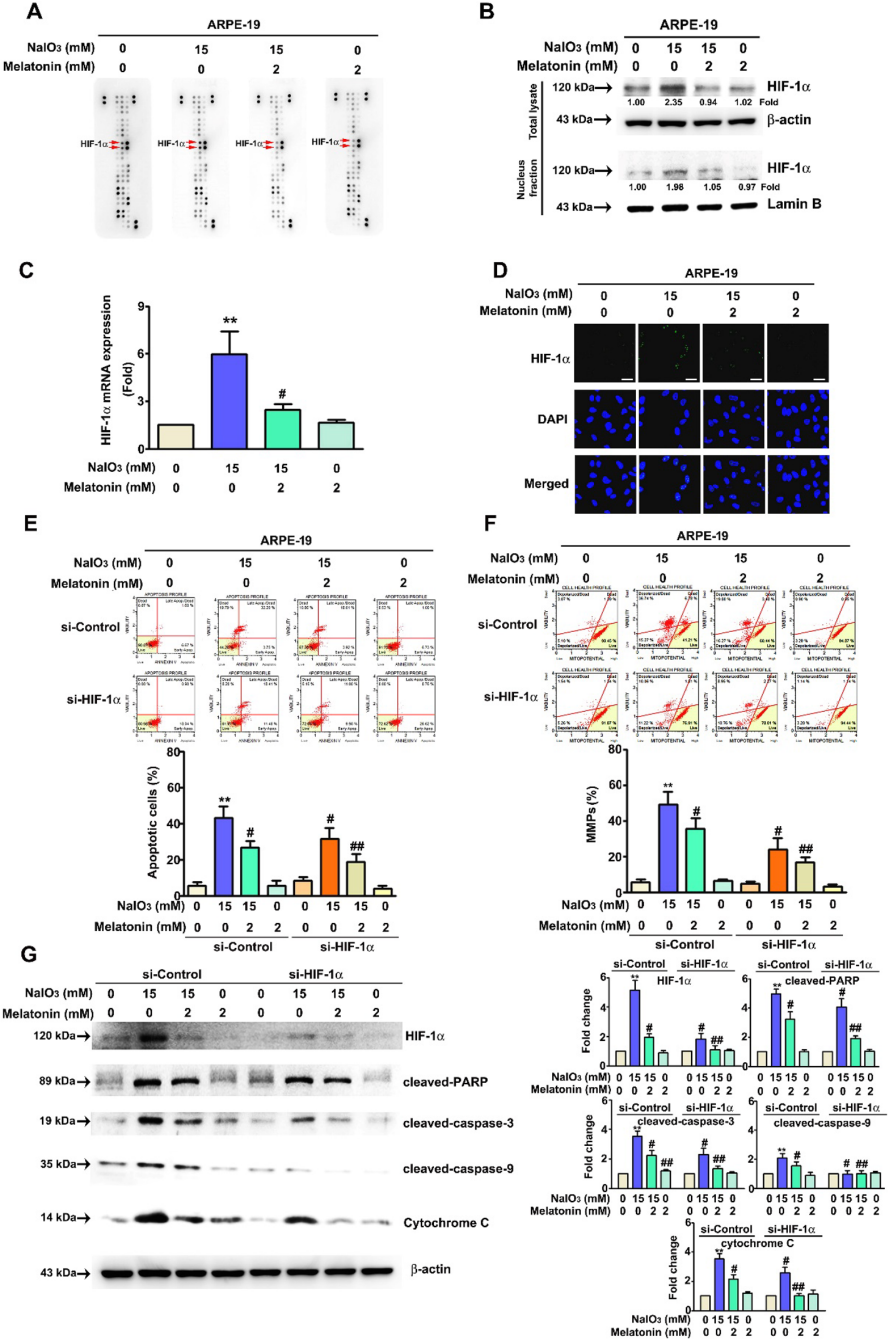
The role played by HIF-1α in NaIO_3_-induced cell apoptosis. **A** Whole-cell lysates of ARPE-19 cells were treated with NaIO_3_ and melatonin and then analyzed with a human apoptosis array. **B** Western blot and quantification of HIF-1α in the nuclear fraction and total lysate of ARPE-19 cells. Lamin B was used as the nuclear fraction control, and β-actin was used as a total cell-lysate control. **C** The mRNA expression of HIF-1α was measured by real-time PCR. GAPDH was used as the internal control. **D** HIF-1α expression was analyzed by immunofluorescence. Scale bar = 50 μm Transfection with HIF-1α siRNA in ARPE-19 cells treated with NaIO_3_ and melatonin. Then, apoptosis **E** and mitochondrial membrane potential **F** were analyzed by flow cytometry assay. **G** Total protein lysate of cells with HIF-1α siRNA transfection was used to explore apoptosis-associated protein levels by western blot assay. β-actin was used as the internal control. All of the data are presented as the mean ± SEM of three independent experiments. ** P < 0.01 compared with si-control group, # P < 0.05 compared with the si-control + NaIO_3_ treatment group, ## P < 0.05 compared with the si-HIF-1α + NaIO_3_ treatment group. MMPs: (upper left): percentage of dead cells with intact mitochondrial membranes; (lower left): percentage of live cells with depolarized mitochondrial membrane

NaIO_3_-induced RPE cell death in a previous study [30]; however, the mechanism through which NaIO_3_ induced ARPE-19 cell death and can be inhibited by melatonin was unclear. To identify the proteins involved in the effects of melatonin and NaIO_3_ treatment, treated cells were collected for further human apoptosis-related protein array analysis. NaIO_3_ treatment increased the protein level of HIF-1α, and cotreatment with melatonin reduced HIF-1α expression (Fig. 3A). Similar results were observed through western blot analysis, nuclear fraction real-time PCR and immunofluorescence assays (Fig. 3B–D). To assess HIF-1α involvement in NaIO_3_-induced cell apoptosis, a transfection assay using short interfering RNA (siRNA) against HIF-1α was performed. Knockdown of HIF-1α in combination with melatonin treatment further suppressed NaIO_3_-induced cell apoptosis and mitochondrial depolarization (Fig. 3E, F). Knockdown of HIF-1α in conjunction with the combination treatment significantly inhibited the expression of cleaved caspase-9, cleaved caspase-3, cleaved PARP, and cytochrome c (Fig. 3G), suggesting a key role for HIF-1α in NaIO_3_-induced cell apoptosis.

### Mechanism of HIF-1α action in melatonin‐inhibited NaIO_3_ induces apoptosis in ARPE-19 cells

**Fig. 4:**
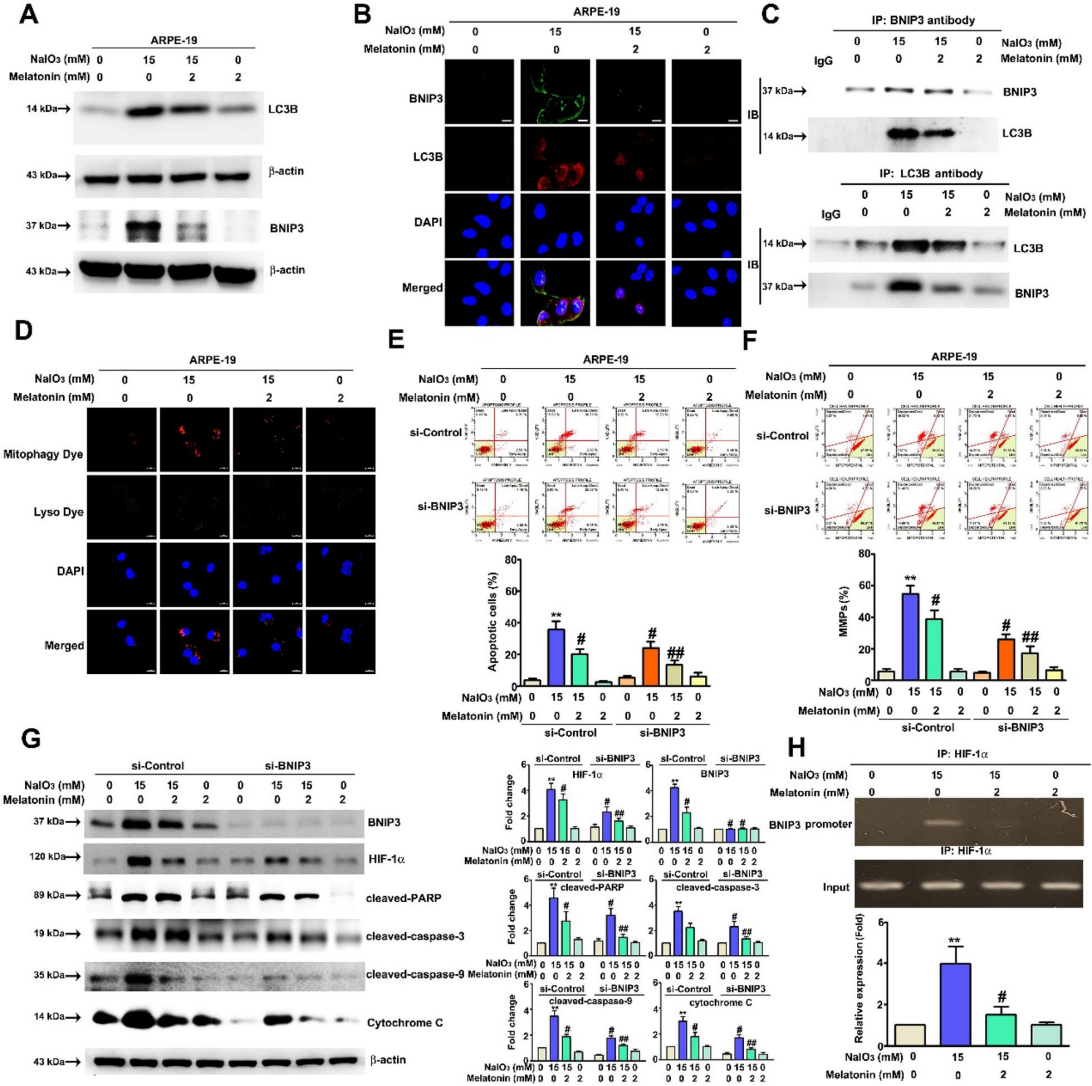
Melatonin attenuated NaIO_3_-induced cell mitophagic apoptosis through HIF-1α/BNIP3/LC3B signaling. **A** The protein levels of the mitophagy markers BNIP3 and LC3B. After treatment, BNIP3 **B** and LC3B **C** expression and mitophagy **D** were detected by immunofluorescence. Scale bar = 50 μm. **E**, **F**, **G** ARPE-19 cells were transfected with BNIP3 siRNA, the cells were collected, and the apoptosis rate and MMP levels were measured. BNIP3 siRNA transfection was validated by western blotting. **H** ChIP assays with anti-HIF-1α antibodies were performed on chromatin extracted from drug-treated ARPE-19 cells. **I**, **J** Immunoprecipitation (IP) data after western blotting were used to investigate the relationship between the BNIP3 and LC3B proteins (β-actin was used as the internal control). All of the data are presented as the mean ± SEM of three independent experiments. ** P < 0.01 compared with the si-control group, # P < 0.05 compared with the si-control + NaIO_3_ treatment group, and ## P < 0.05 compared with the si-BNIP3 + NaIO_3_ treatment group

HIF-1α activation is necessary for triggering NaIO_3_-induced apoptosis, and HIF-1α is considered to be the transcription factor of BNIP3 [31]. In addition, BNIP3 has been demonstrated to be involved in mitophagy signaling [32], resulting in mitophagy and combined activation of LC3B on the mitochondrial membrane and leading to enhanced signaling downstream. The expression of BNIP3 and LC3B was detected after cotreatment was administered. The protein levels of BNIP3 and LC3B were similar to those of HIF-1α (Fig. 4A). The results of immunofluorescence staining showed that melatonin suppressed BNIP3 and LC3B activation after NaIO_3_ treatment (Fig. 4B). An immunoprecipitation assay was performed to examine the interaction between BNIP3 and LC3B. The immunoblot results suggested that melatonin decreased the binding ability of BNIP3 and LC3B, which had been enhanced via NaIO_3_ treatment (Fig. 4C). A mitophagy detection kit was used to quantify mitophagy activation after treatment. Melatonin effectively reduced NaIO_3_ treatment-induced accumulation of mitophagosomes in ARPE-19 cells (Fig. 4D). To assess mitophagy activity through immunofluorescence, a mitochondria-targeted red fluorescent protein Keima (mt Keima)-Parkin [33] was used. We observed that NaIO_3_-induced PINK1-dependent mitophagy, which was rescued in melatonin-treated cells (Additional file 1: Fig. S2). To confirm the function of BNIP3 in NaIO_3_-induced cell apoptosis, we transfected BNIP3 siRNA into cells. Knockdown of BNIP3 combined with melatonin treatment profoundly suppressed NaIO_3_-induced cell apoptosis and mitochondrial depolarization. Western blotting demonstrated that knockdown of BNIP3 combined with melatonin treatment inhibited the expression of cleaved-caspase-9, cleaved-caspase-3, cleaved-PARP, and cytochrome c (Fig. 4E–G). Chromatin immunoprecipitation was performed to confirm the relationship between HIF-1α and BNIP3. Melatonin reduced promoter binding by HIF-1α, which had been enhanced by NaIO_3_ treatment (Fig. 4H). Taken together, these results suggest that melatonin inhibits NaIO_3_-induced ARPE-19 cell apoptosis via suppression of the HIF-1α/BNIP3-LC3B axis in mitophagy signaling.

### Effect of melatonin inhibits NaIO_3_-induced ROS-mediated mitophagy signaling pathway

**Fig. 5:**
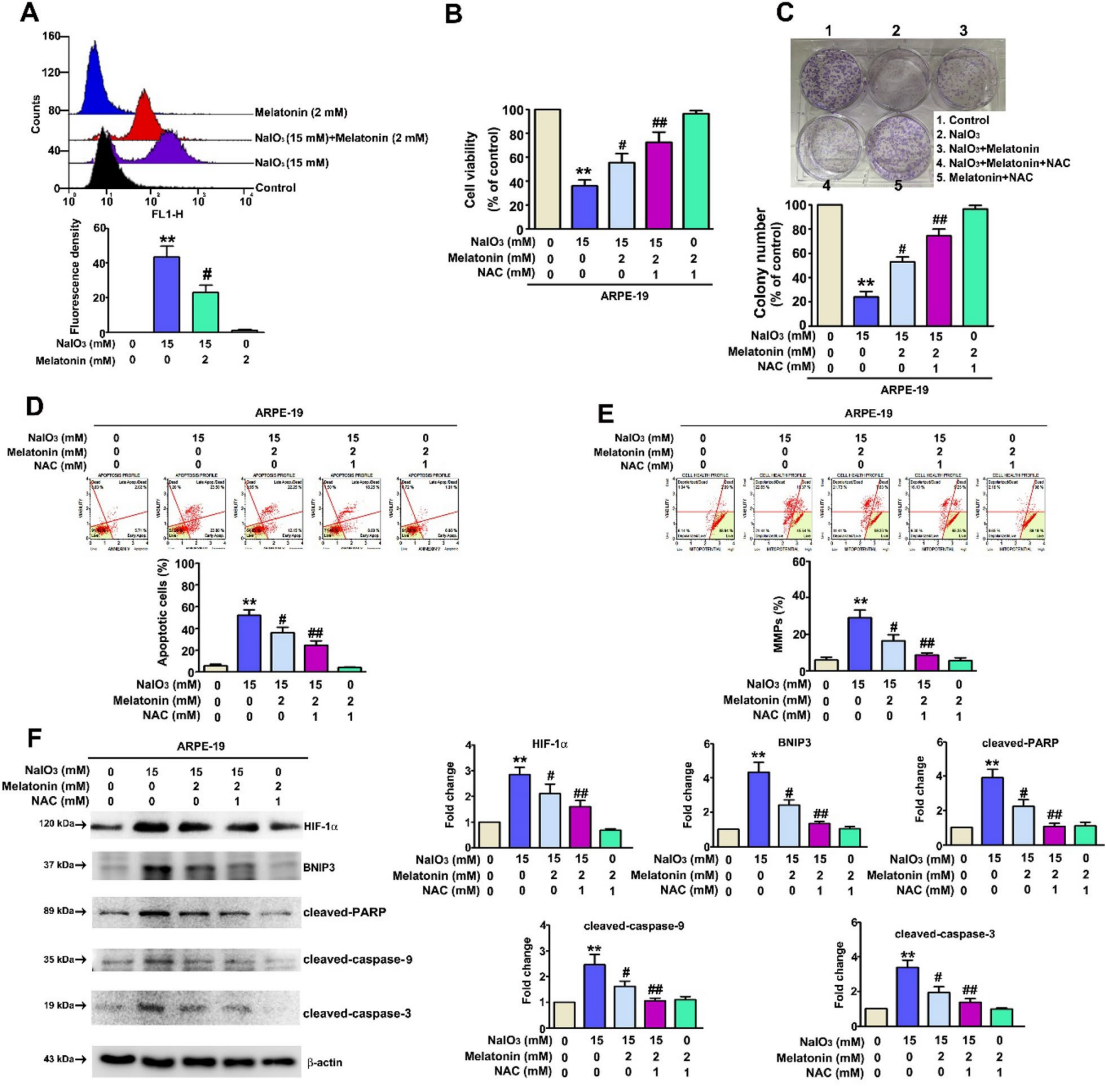
Effects of treatment with melatonin or NAC on NaIO_3_-induced ROS production in ARPE-19 cells. **A** ARPE-19 cells were cotreated with melatonin and NaIO_3_ for 24 h. Flow cytometry data were obtained using a DCFH-DA dye. **B**, **C** The cell proliferation rate was determined by MTT assay and colony formation assay. **D**, **E** ARPE-19 cells were cotreated with the ROS inhibitor NAC and melatonin and NaIO_3_. Cells were then collected, and the apoptosis rate and MMP levels were measured by flow cytometry. **F** Western blotting was performed to determine the effect of NAC treatment (β-actin was used as the internal control). All of the data are presented as the mean ± SEM of three independent experiments. **P < 0.01 compared with the control group, # P < 0.05 compared with the NaIO_3_ treatment group, and ## P < 0.05 compared with the NaIO_3_ + melatonin treatment group. MMPs: (upper left): percentage of dead cells with intact mitochondrial membranes; (lower left): percentage of live cells with depolarized mitochondrial membranes

ROS are considered to be causes of AMD [33]. A NaIO_3_ cell model was reported to promote the ROS accumulation [28]. Performing a staining assay with the fluorescent ROS probe DCFH-DA, NaIO_3_ combined with melatonin was found to cause a significant decrease in ROS production compared with the effect of NaIO_3_ treatment alone (Fig. 5A); therefore, we used the ROS inhibitor N-acetylcysteine (NAC) combined with NaIO_3_ and melatonin to examine the role played by ROS. NAC in combination with NaIO_3_ and melatonin significantly protected ARPE-19 cells against NaIO_3_-induced injury (Fig. 5B, C). NAC combined with melatonin suppressed NaIO_3_-induced cell apoptosis and mitochondrial depolarization (Fig. 5D, E). The western blot results demonstrated that NAC combined with melatonin inhibited the expression of cleaved-caspase-9, cleaved-caspase-3, cleaved-PARP, BNIP3, and LC3B (Fig. 5F). In addition, hypoxia causes a gradient of oxidative stress mediated by H_2_O_2_, promoting various ocular diseases, such as retinopathy and age-related macular degeneration [34, 35]. The results showed that ARPE-19 cells treated with H_2_O_2_ exhibited significantly decreased cell viability; this effect was rescued by melatonin treatment (Additional file 1: Fig. S3A). Additionally, melatonin suppressed H_2_O_2_-induced (Additional file 1: Fig. S3B) and apoptosis (Additional file 1: Fig. S3C). However, the western blot findings suggested that melatonin significantly reduced the H_2_O_2_-induced expression of HIF-1α, BNIP3, and LC3B in ARPE-19 cells (Additional file 1: Fig. S3D). H_2_O_2_ combined with melatonin suppressed H_2_O_2_-induced mitophagy (Additional file 1: Fig. S4A) and mitochondrial Keima-Red expression in ARPE-19 cells (Additional file 1: Fig. S4B). These results suggested that melatonin inhibits NaIO_3_-induced ROS-mediated mitophagy via HIF-1α targeting that inhibits BNIP3/LC3B signaling pathway.

### Protective effects of melatonin on retinal degeneration in NaIO_3_-treated mice

**Fig. 6:**
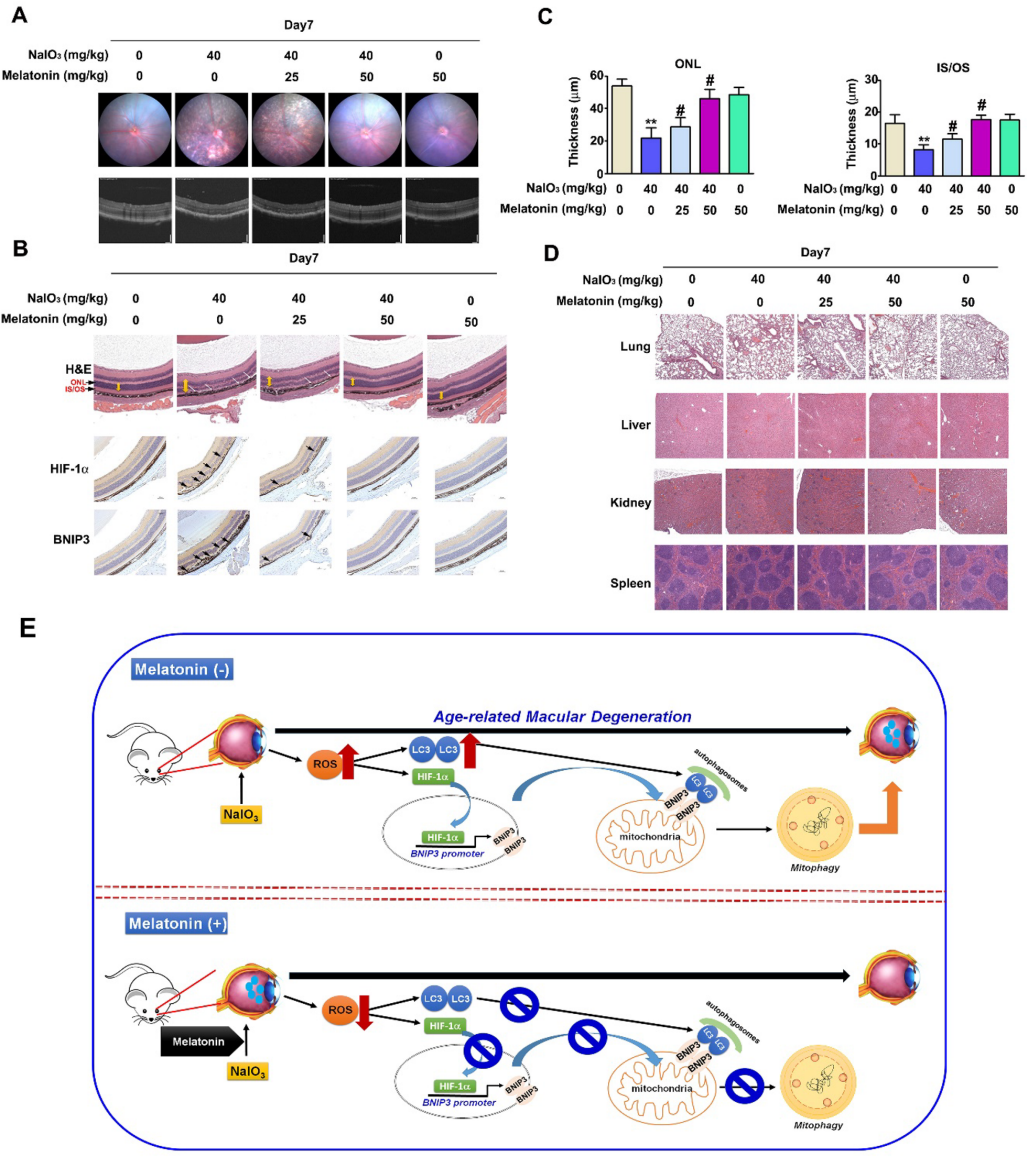
The effects of melatonin on retinal degeneration in NaIO_3_-treated mice. C57BL/6 mice were pretreated with melatonin (25, 50 mg/kg) for 7 days and cotreated with NaIO_3_ via intravenous injection. **A** After intravenous injection, changes in the optical coherence tomographic (OCT) images in the day 7-group mice were collected using a Micron IV camera. **B** Retinal histology for day 7 mice was detected by H&E staining and IHC staining. **C** The lengths of the ONL and IS/OS were quantified on the basis of retinal histology data. **D** Normal organ tissues were examined by H&E staining. **E** Proposed mechanism by which melatonin alleviates NaIO_3_-induced ROS stress-mediated mitophagy through HIF-1α/BNIP3 modulation of LC3 expression in vitro and in vivo. All of the data are presented as the mean ± SEM of three independent experiments. **P < 0.01 compared with the control group and # P < 0.05 compared with the NaIO_3_ treatment group

To confirm our results of cell NaIO_3_-induced damage observed in vitro, a retinal degeneration mouse model was established. Oral treatment of the mice with melatonin (25 or 50 mg/kg) for 7 days was followed by NaIO_3_ injection into the tail vein (40 mg/kg) and then oral treatment with melatonin was reinitiated and maintained for 7 days. After treatment, fundus photographs and optical coherence tomography (OCT) were performed to observe the thickness of the whole retina, inner nuclear layer (INL) and outer nuclear layer (ONL). Significant pigmentary changes in the RPE layer and loss of retinal lamination were observed in the NaIO_3_ group (Fig. 6A). However, photographs and OCT taken after high-dose melatonin treatment revealed a fundus similar to that of the control group. In addition, Fig. 6B displays a tissue slice stained with hemoxylin and eosin (H&E) and immunohistochemical dye suggested that the high-dose melatonin group exhibited reduced NaIO_3_-induced overexpression of BNIP3 and HIF-1α in the RPE layer (Fig. 6B). The thicknesses of the ONL, IS and OS were then quantified (Fig. 6C). Melatonin treatment restored retinal thickness, and no indication of damage to the lung, liver, heart, kidney, or spleen was observed (Fig. 6D). These results suggest that oral treatment with melatonin effectively reduced NaIO_3_-induced retinal injury.

## Discussion

AMD remains a major cause of blindness in the elderly population. An effective treatment to reduce disease progression has not been identified to date [36]. Melatonin is a naturally occurring and important hormone that has been reported to protect RPE cells from oxidative stress [37]. Melatonin has been shown to inhibit EGF-induced proliferation and motility of ARPE-19 cells by activating the AKT/mTOR pathway [20]. We explored the protective effect of melatonin on ARPE-19 cells and RPE cell after NaIO_3_‐induced cell injury to further identify treatment options for AMD. First, we found that a low dose of melatonin effectively reduced cell death through inactivation of apoptosis signaling in NaIO_3_-AMD cell models. In addition, knockdown of BNIP3 and HIF-1α directly enhanced the efficiency of melatonin treatment. Melatonin disrupted the binding between BNIP3 and HIF-1α, resulting in inhibition of NaIO_3_-induced mitophagy. Upstream HIF-1α activation and ROS production were decreased after treatment, suggesting that melatonin regulated NaIO_3_-induced cell apoptosis in RPE cells via ROS activation. In an AMD animal model, melatonin treatment alleviated NaIO_3_-induced retinal dysfunction and RPE cell injury (Fig. 6E); thus, melatonin may be a potential treatment for patients with AMD.

HIF-1α is a critical protein that mediates adaptive responses to hypoxia by regulating the expression of various genes [38]. A recent study reported that HIF-1α can block mitochondrial respiration and electron transport chain (ETC) activity by regulating miR-210 expression in various cell types [39]. Furthermore, hypoxia-induced mitochondrial autophagy is required for HIF-1α targeting BNIP3 [40] and LON [41] expressions. Suppression of the mitochondrial ROS/HIF-1α pathways through direct inhibition of HIF-1α induced ETC complex I dysfunction and metabolic pathways in leukemia and lymphoma [42]. Autophagy may be involved in the mechanism of oxidative stress activation. Since the transport of cargo was compromised in degenerative RPE cells in patients with AMD, the activation of the autophagy pathway may have been delayed or blocked, leading to increased oxidative stress and causing irreversible injury to RPE cells [43]. Autophagy is important to the metabolism of RPE cells. Knockdown of the ATG7 or BECN1 gene in H_2_O_2_-treated ARPE-19 cells increased ROS generation. In addition, rapamycin treatment promoted autophagy signaling, causing decreased ROS production [44]. These results suggest that autophagy confers RPE cell resistance to oxidative stress and may alleviate AMD.

Mitophagy is a kind of autophagy that is characterized by removal damaged mitochondria through selective pathways. Recent studies have reported that aging promotes the accumulation of damaged mitochondria since aging decreases the efficacy of mitophagy [45], promoting the accumulation of mitochondrial ROS and oxidative damage. A previous stud showed that aged RPE cells exhibit more significant mtDNA damage [23]. These results suggest that mitophagy plays an essential role in AMD. Additionally, melatonin has been reported to protect cells from oxidative damage via mitophagy repression [46], suggesting that melatonin regulates ROS and mitophagy. Melatonin effectively reduced retinal injury in AMD, as demonstrated by previous in vivo animal and in vitro RPE cell models [47, 48]. Melatonin protected RPE cells via inactivation of oxidative stress-induced apoptosis and activation of autophagy [49]. The molecular mechanisms related to AMD include oxidative stress and inflammation, but the underlying mechanisms remain unclear. In the present study, a human apoptosis protein array analysis demonstrated that HIF-1α is a vital protein associated with angiogenesis, and its abundance increased after NaIO_3_ treatment; when cotreated with melatonin, the expression of HIF-1α was decreased.

Additionally, BNIP3 expression changed in parallel with HIF-1α expression changes, since HIF-1α regulated BNIP3 levels via transcription [31]. Moreover, based on the current study results, HIF-1α/BNIP3 signaling plays an important role in oxidative stress-induced mitophagic cell death. Therefore, HIF-1α/BNIP3 might be a target in AMD treatment. Additionally, decreased HIF-1α expression in NaIO_3_-treated ARPE-19 cells was associated with BNIP3 knockdown, which was particularly intriguing. Upregulation of HIF-1α induced apoptosis of the hUSLF cells through the increased expression of BNIP3 [50]. Other reports demonstrated that knockdown of BNIP3 markedly attenuated HIF-1α inhibition of human HCN-1A cell apoptosis and induced autophagic cell survival [51].

## Conclusion

Our study demonstrates that melatonin reduced NaIO_3_-induced ROS-mediated mitophagy in ARPE-19 cells through the suppression of HIF-1α targeting of BNIP3 signaling. HIF-1α/BNIP3 should be considered a novel therapeutic target for AMD.

## Methods

### Reagents and antibodies

Sodium iodate (NaIO_3_) was purchased from Thermo Fisher Scientific (Tewksbury, MA, USA). MTT powder (M5655), cobalt(II) chloride (232,696), hydrogen peroxide solution (31,642), Giemsa (GS500), DAPI (D9542), N-acetyl-L-cysteine (A7250) and melatonin (M5250) were purchased from Sigma (St. Louis, MO, USA). A Human Apoptosis Array Kit (ARY009) was purchased from R&D Systems, Inc. (Minneapolis, MN, USA). A Muse® Annexin V & Dead Cell Kit (MCH100105) and Muse® MitoPotential Kit (MCH100110) were purchased from Luminex Corporation (Austin, TX, USA). DCFH-DA was purchased from AAT Bioquest, Inc. (Sunnyvale, CA, USA). An AllPure Mammalian Mitochondria Isolation Kit for Cultured Cells (ABTGDE401) was purchased from Allbio Science. Fetal bovine serum (FBS, SH30071.03), penicillin‒streptomycin solution (100X; SV30010) and trypsin 0.25% (SH30042.01) were purchased from HyClone (Logan, UT, USA). Antibodies against BNIP3 (sc-56167), Bcl-2 (sc-492), cytochrome c (sc-13156), β-actin (sc-69879), Lamin B (sc-6216), siRNA-BNIP3 (sc-37451) and siRNA-HIF-1α (sc-35561) for use in Western blotting were purchased from Santa Cruz Biotechnology (Santa Cruz, CA, USA); antibodies against cleaved-PARP (#9542), cleaved caspase3 (#9668), cleaved caspase9 (#9508) and Bax (#5023) were purchased from Cell Signaling Technology (Beverly, MA, USA); an antibody against HIF-1α (NB100-105) was purchased from Novus Biologicals (Centennial, CO, USA); and antibodies against goat anti-rabbit IgG (AP132P) and goat anti-mouse IgG (AP124P) were purchased from Merck Millipore (CEDEX, France).

### Cell culture and drugs treatment

The ARPE-19 human retinal pigment epithelia cell line was obtained from the Bioresources Collection and Research Center (BCRC), Food Industry Research and Development Institute (Hsinchu, Taiwan). ARPE-19 cells were cultured in Dulbecco’s modified Eagle’s medium/nutrient mixture F-12 Ham (DMEM/F12) containing 10% FBS and 1% penicillin/streptomycin antibiotic at 37 °C with 5% CO_2_. For drug treatment, ARPE-19 cells were treated with melatonin (2 mM) in the absence or presence of NaIO_3_ (15 mM). For inhibitor treatment, ROS inhibitor NAC (1 mM) was pre-treated with melatonin (2 mM) for 2 h, and then added with or without of NaIO_3_ (15 mM).

### Cell viability assay

Cell viability was detected by MTT assay. ARPE-19 cells were seeded in 24-well culture plates (4 × 10^4^ cells/well) and treated with melatonin and NaIO_3_ or NAC for 24 h. Fresh medium containing MTT (0.5 mg/ml) was used to incubate the treated cells for 4 h, and then, 0.8 ml of isopropanol was added to dissolve the purple formazan. Absorbance was measured at 570 nm with a microplate reader (Labsystems, Helsinki, Finland).

### Colony formation assay

ARPE-19 cells were seeded in 6-well culture plates (5 × 10^3^ cells/well) and treated with melatonin (0.5, 1 and 2 mM) with or without NaIO_3_ (15 mM) for 2 weeks. The colonies were washed twice with PBS, fixed with methanol and stained with PBS containing 5% v/v Giemsa solution for 4 h. Colonies were measured and photographed.

### Annexin-V/PI assay and measurement of mitochondrial membrane potential (MMP)

For Annexin-V/PI detection and MMP analysis, ARPE-19 cells were seeded in 6 cm dishes (4 × 10^5^ cells) and treated with melatonin (1 and 2 mM) with or without NaIO_3_ (15 mM) for 24 h. After drug treatment, the cells were collected and stained with a Muse^®^ Annexin V & Dead Cell Kit (cell apoptosis assay). Y-axis presents the cell viability (%) of normal healthy cells (LL), early and apoptotic cells (LR/UR) and necrotic cells (UL). Detection of the mitochondrial membrane potential (MMP) in ARPE-19 cells by used the Muse^®^ MitoPotential Kit (MMP assay) were detected by using Muse^®^ Cell Analyzer (Millipore, Hayward, CA, USA). (Upper Left): % of dead cells with intact mitochondrial membrane; (Low Left): % of live cells with depolarized mitochondrial membrane.

### Determination of the ROS

First, ARPE-19 cells were seeded in 6 cm dishes (4 × 10^5^ cells) and treated with melatonin (2 mM) with or without NaIO_3_ (15 mM) for 24 h. ROS production was detected using DCFH-DA (10 μM) staining at 37 °C for 30 min, and the cells were collected and analyzed by FACSCalibur flow cytometry (BD FACSCalibur, Becton Dickinson Co., Franklin Lakes, NJ, USA).

### Monitoring of mitophagy

Treatment cells were cultured on 8‐well Lab‐Tek Chambered Coverglass (2 × 10^4^ cells) for 24 h, washed with PBS, fixed with 4% paraformaldehyde for 10 min, and permeabilized with PBS containing 0.1% Triton X‐100 for 10 min. DAPI was incubated with 2% bovine serum albumin at room temperature for 2 h, and the cells were stained using a Dojindo Mitophagy Detection Kit (Dojindo EU GmbH, Munich, Germany). After staining, the cells were visualized with a Zeiss LSM 510 META confocal microscope (Heidelberg, Germany) and analyzed. Keima was used for mitochondria detection, and cells were transfected with a pMitophagy Keima-Red mPark2 plasmid using TurboFect transfection reagent for 6 h; the medium was then replaced with fresh medium, and the cells were incubated for 18 h. The cells were then treated with melatonin and NaIO_3_. After staining, the change in mt-Keima fluorescence was analyzed with a Zeiss LSM 510 META confocal microscope (Heidelberg, Germany) according to the manufacturer’s protocols.

### Human apoptosis array analysis

ARPE-19 cells were seeded in 10 cm dishes (1.2 × 10^6^ cells) and treated with melatonin and NaIO_3_ for 24 h. After drug treatment, the cells were lysed using lysis buffer containing protease inhibitor, sonicated and centrifuged. The supernatant was collected for the analysis of apoptosis or antiapoptotic protein levels using a Human Apoptosis Array Kit (ARY009).

### Mitochondria lysate and nuclear fraction preparation

ARPE-19 cells were seeded in 10 cm dishes (1.2 × 10^6^ cells) and treated with melatonin or a combination of the aforementioned drugs for 24 h. After washing twice with PBS, mitochondria and nuclear lysates were isolated using an AllPure Mammalian Mitochondria Isolation Kit and Nuclear Protein Isolation Kit following the respective manufacturer’s instructions.

### Cell lysate preparation and Western blot analysis

After drug treatment, cell pellets were lysed with lysis buffer containing protease inhibitor and sonicated on ice. Protein samples were centrifuged for 30 min at 13,000 rpm, and the concentration was measured by the Bradford method (Bio–Rad). Equal amounts (20 μg) of protein sample were separated by 10–12% SDS‒PAGE for 2 h and transferred to PVDF membranes for 2 h. The membranes were blocked with Tris‐buffered saline containing 0.1% v/v Tween‐20 (TBST) containing 5% v/v nonfat milk for 1 h. The primary antibodies were incubated with the membrane overnight at 4 °C, and the secondary antibodies were incubated with the membrane at room temperature. The results were detected with an Luminescent Image Analyzer LAS‐4000 mini.

### siRNA transfection

ARPE-19 cells were cultured on 6 cm dishes (2.5 × 10^5^ cells) for 24 h. The cells were incubated with siRNA and Lipofectamine RNAiMAX Transfection Reagent for 6 h, the medium was replaced, and the cells were incubated for 18 h. Then, the cells were treated with melatonin and NaIO_3._ The sequences of small inhibitory RNAs (siRNAs) specifically targeting BNIP-3 (siBNIP-3; a pool of sc-37451A (sense: GAACUGCACUUCAGCAAUAtt; antisense: UAUUGCUGAAGUGCAGUUCtt), sc-37451B (sense: CCAUAGCAUUGGAGAGAAAtt, antisense: UUUCUCUCCAAUGCUAUGGtt), and sc-37451C (sense: GAAGGCACCUACUCAGUAUtt, antisense: AUACUGAGUAGGUGCCUUCtt)) and HIF-1α (si-HIF-1α; sense: CUGAUGACCAGCAACUUGAtt; antisense: UCAAGUUGCUGGUCAUCAGtt), as well as a scrambled control siRNA, were constructed by and obtained from Santa Cruz Biotechnology (Santa Cruz, CA, USA).

### Isolation of RNA and real-time qRT-PCR

The total RNA of the ARPE-19 cells was extracted using TRIzol reagent (Invitrogen, Carlsbad, CA), and the cDNA was reverse transcribed using GoScript™ Reverse Transcription Mix (Promega Corporation). Gene expression was detected with GoTaq qPCR Master Mix reagents (Promega Corporation) in an ABI PRISM 7700 real-time PCR system (Applied Biosystems, Foster City, CA, USA). The real-time PCR primer pairs were as follows: for GAPDH, 5′-CATCATCCCTGCCTCTACTG-3′ (forward) and 5′-GCCTGCTTCACCACCTTC-3′ (reverse); for BNIP3, 5′-GCCATCGGATTGGGGATCTAT-3′ (forward) and 5′-GCCACCCCAGGATCTAACAG-3′ (reverse); and for HIF-1α, 5′-GAACGTCGAAAAGAAAAGTCTCG-3′ (forward) and 5′-CCTTATCAAGATGCGAACTCACA-3’ (reverse). GAPDH was used as the internal control. All of the gene levels were normalized to the level of the GAPDH bene, and fold change was calculated by the 2^−ΔΔCt^ method.

### Chromatin immunoprecipitation (ChIP)

ARPE-19 cells were seeded in 10 cm dishes (1.2 × 10^6^ cells) and treated with melatonin and NaIO_3_ for 24 h. Cells were crosslinked with 4% paraformaldehyde for 10 min and incubated with 125 mM glycine for 5 min at room temperature. Then, the lysates were sonicated and immunoprecipitated with antibody against HIF-1α or with mouse IgG. Samples were incubated at 65 °C overnight, RNase was added for 1 h at 37 °C, and proteinase K was added for 2 h at 45 °C. After purification, DNA as dissolved in 20 μl nuclease-free water. The ChIP primers used for the real-time PCR were as follows: for BNIP3, 5′- CTTCCC TGCACGTCCTCAC-3′ (forward) and 5′-CCGGGTTCTCCTTTGAAGGG-3′ (reverse). The data were collected using an ABI PRISM 7700 real-time PCR system.

### Immunofluorescence staining

ARPE‐19 cells were seeded in 8‐well Lab‐Tek Chambered Coverglass (2 × 10^4^ cells) and treated with melatonin and NaIO_3_ for 24 h. Cells were washed with PBS, fixed with 4% paraformaldehyde for 10 min, permeabilized with PBS containing 0.1% Triton X‐100 for 10 min, and blocked with 2% bovine serum albumin for 2 h. Primary antibodies against HIF-1α and BNIP3 were incubated in 2% bovine serum albumin at 4 °C overnight, and secondary antibodies were incubated in 2% bovine serum albumin at room temperature for 2 h. DAPI reagent was used for counterstaining the cell nucleus. The data were visualized with a Zeiss LSM 510 META confocal microscope (Heidelberg, Germany) and analyzed.

### In vivo animal model and immunohistochemistry analysis

This protocol of the animal experiment was approved by the Institutional Animal Care and Use Committee of Chung Shan Medical University (IACUC number: 2400). 5-week-old male C57BL/6JNarl mice were obtained from the National Laboratory Animal Center (Taipei, Taiwan). Before NaIO_3_ treatment, the mice were assigned to a 7-day group, and this group was divided into 5 subgroups (n = 5 per subgroup) and treated orally with melatonin (25 and 50 mg/kg) for 7 days. Melatonin was administered orally before the room lights were switched off for 1 h. After melatonin pretreatment for 7 days, NaIO_3_ was injected into the tail vein (40 mg/kg), and then, the mice were given melatonin orally for the final 7 days of the experiment. After melatonin treatment mice in the 7-day group were subjected to OCT imaging and sacrificed. Lung, liver, spleen, kidney and eye tissues were harvested, fixed in formalin, and processed for H&E staining. The retina in the eyes was analyzed by BNIP3 and HIF-1α staining.

### In vivo mice optical coherence tomography

Mice were anesthetized, and then, the pupils were dilated using a drop of 1.0% tropicamide (Alcon Laboratories, Inc.). Photography and fluorescein angiography of the retina in each eye were performed with a Micron IV camera (Phoenix Research Laboratories, Inc.). Optical coherence tomography images were taken with an image-guided tomographer (Micron IV-OCT2; Phoenix Research Laboratories, Inc.).

### Statistical analysis

All of the data as are presented as the mean ± SEM of at least three independent experiments. The significance of the differences between datasets was assessed by t test or one‐way ANOVA (GraphPad Prism 6). Differences were considered significant when P < 0.05 or P < 0.01.

## Supplementary Information

**Additional file 1: Figure S1.** Mitochondria cell lysate from ARPE-19 cells were treated with NaIO_3_ and melatonin, then analyzed using a western blot and quantification of the mitochondria fraction of Cytochrome C expression. COXIV as mitochondria control. All of the data are presented as the mean ± SEM of three independent experiments. **Figure S2.** ARPE-19 cells were transfected with mitochondria-targeted reg fluorescent protein Keima (mt-Keima), and treated with or without melatonin in NaIO_3_-treated cells. Representative images of Keima-Red by immunofluorescence assay. Scale bars, 50 μm. Results are representative of at least three independent experiments. **Figure S3.** ARPE-19 cells were co-treated with H_2_O_2_ (1 mM) and melatonin (2 mM) for 24 h, (A) cell viability was measured using an MTT assay. (B) Flow cytometry data was detected using a DCFH-DA dye. (C) Cell apoptotic cells were detected with an Annexin-V/PI staining by flow cytometry. (D) The protein expression of HIF-1a, BNIP3 and LC3B were determined with western blotting, β-actin was used as the internal control. All of the data are presented as the mean ± SEM of three independent experiments. **, P < 0.01 compared with control and #, P < 0.05 compared with H_2_O_2_. **Figure S4.** (A) ARPE-19 cells were co-treated with H_2_O_2_(1 mM) and melatonin (2 mM) for 24 h, then incubated with mitophagy dye for 15 mins by immunofluorescence assay. (B) Transfected with mitochondria-targeted fluorescent protein Keima (mt-Keima), and treated with or without melatonin (2 mM) in H_2_O_2_-treated ARPE19 cells. Representative images of Keima-Red were detected by immunofluorescence assay. Scale bars, 50 μm.

## Data Availability

The data that support the findings of this study are available from the corresponding author upon reasonable request.

## Abbreviations

AMD: Age-related macular degeneration
BNIP3: BCL2 and adenovirus E1B 19-kDa-interacting protein 3
ChIP: Chromatin immunopecipitation
DAPI: 4',6-Diamidino-2-phenylindole
HIF-1α: Hypoxia-inducible factor-1α
IHC: Immunohistochemistry
INL: Inner nuclear layer
LC3B: MAP1LC3B
MMP: Mitochondrial membrane potential
MTT: 3-(4,5-Dimethylthiazol-2-yl)-2,5-diphenyltetrazolium bromide
NAC: N-acetyl-L-cysteine
NaIO_3_: Sodium iodate
OCT: Optical coherence tomography
ONL: Outer nuclear layer
qRT-PCR: Quantitative real-time reverse transcription polymerase chain reaction
ROS: Reactive oxygen species
siRNA: Small interfering RNA
