# Supplementary material for: Melatonin inhibits NaIO3-induced ARPE-19 cell apoptosis via suppression of HIF-1α/BNIP3-LC3B/mitophagy signaling
Source: Cell Biosci. 2022 Aug 19;12:133. doi: 10.1186/s13578-022-00879-3 (PMC9389659; doi:10.1186/s13578-022-00879-3)
Supplement: Supplementary file 1 — Additional file 1: Figure S1. Mitochondria cell lysate from ARPE-19 cells were treated with NaIO3 and melatonin, then analyzed using a western blot and quantification of the mitochondria fraction of Cytochrome C expression. COXIV as mitochondria control. All of the data are presented as the mean ± SEM of three independent experiments. Figure S2. ARPE-19 cells were transfected with mitochondria-targeted reg fluorescent protein Keima (mt-Keima), and treated with or without melatonin in NaIO3-treated cells. Representative images of Keima-Red by immunofluorescence assay. Scale bars, 50 μm. Results are representative of at least three independent experiments. Figure S3. ARPE-19 cells were co-treated with H2O2 (1 mM) and melatonin (2 mM) for 24 h, (A) cell viability was measured using an MTT assay. (B) Flow cytometry data was detected using a DCFH-DA dye. (C) Cell apoptotic cells were detected with an Annexin-V/PI staining by flow cytometry. (D) The protein expression of HIF-1a, BNIP3 and LC3B were determined with western blotting, β-actin was used as the internal control. All of the data are presented as the mean ± SEM of three independent experiments. **, P < 0.01 compared with control and #, P < 0.05 compared with H2O2. Figure S4. (A) ARPE-19 cells were co-treated with H2O2(1 mM) and melatonin (2 mM) for 24 h, then incubated with mitophagy dye for 15 mins by immunofluorescence assay. (B) Transfected with mitochondria-targeted fluorescent protein Keima (mt-Keima), and treated with or without melatonin (2 mM) in H2O2-treated ARPE19 cells. Representative images of Keima-Red were detected by immunofluorescence assay. Scale bars, 50 μm. [file 13578_2022_879_MOESM1_ESM.docx]

Melatonin inhibits NaIO3-induced ARPE-19 cell apoptosis via suppression of HIF-1α/BNIP3-LC3B/mitophagy signaling

**Kai Wang, Yong-Syuan Chen, Hsiang-Wen Chien, Hui-Ling Chiou, Shun-Fa Yang, Yi-Hsien Hsieh**

**Additional file 1: Figure S1**

**
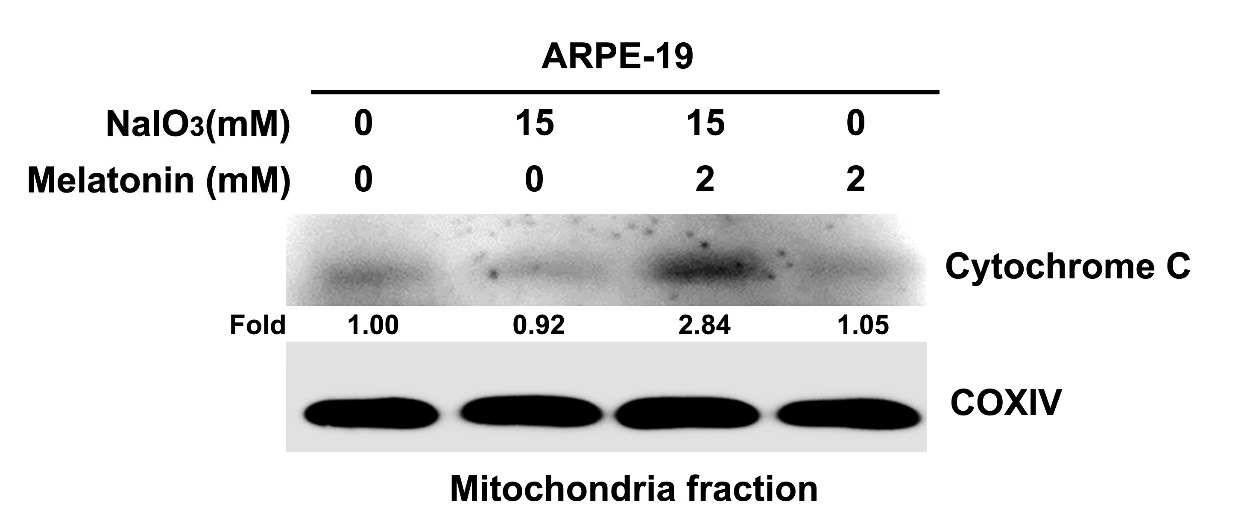
**

**Additional file 1: Figure S1**: Mitochondria cell lysate from ARPE-19 cells were treated with NaIO_3_ and melatonin, then analyzed using a western blot and quantification of the mitochondria fraction of Cytochrome C expression. COXIV as mitochondria control. All of the data are presented as the mean ± SEM of three independent experiments.

**Additional file 1: Figure S2**

**
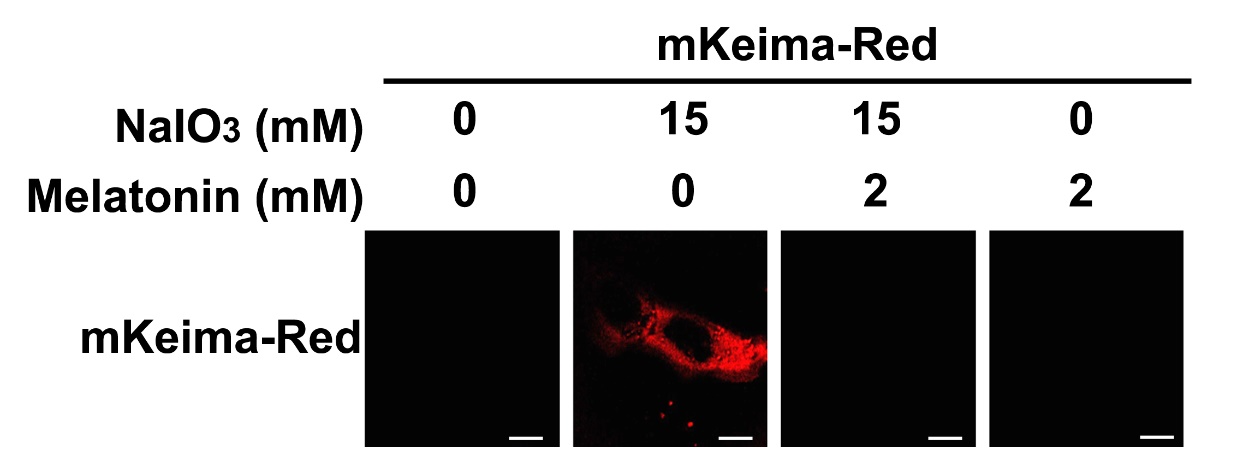
**

**Additional file 1: Figure S2**: ARPE-19 cells were transfected with mitochondria-targeted reg fluorescent protein Keima (mt-Keima), and treated with or without melatonin in NaIO3-treated cells**.** Representative images of Keima-Red by immunofluorescence assay. Scale bars, 50 μm. Results are representative of at least three independent experiments.

**Additional file 1: Figure S3**


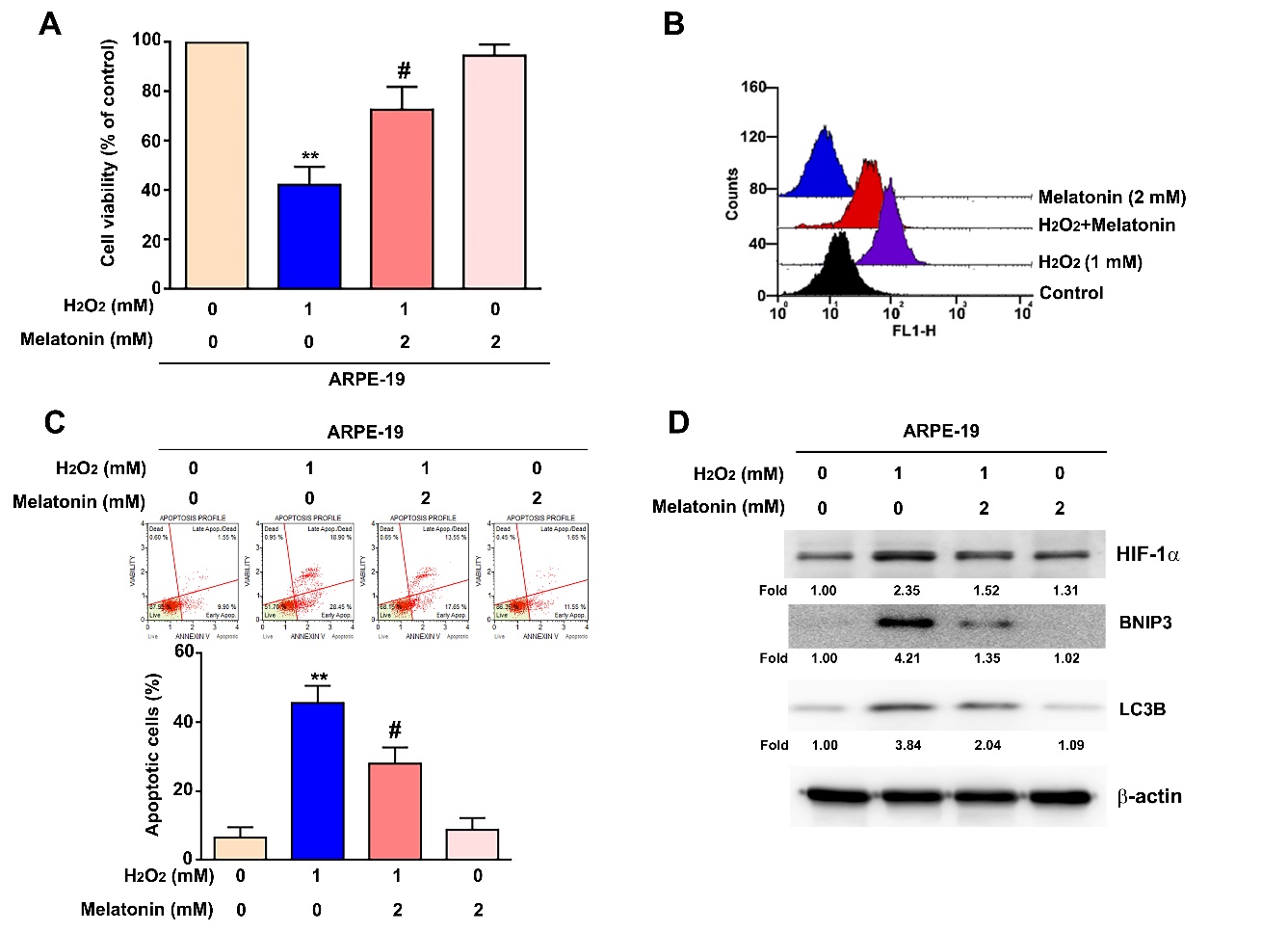


**Additional file 1: Figure S3**: ARPE-19 cells were co-treated with H_2_O_2_ (1 mM) and melatonin (2 mM) for 24 h, (A) cell viability was measured using an MTT assay. (B) Flow cytometry data was detected using a DCFH-DA dye. (C) Cell apoptotic cells were detected with an Annexin-V/PI staining by flow cytometry. (D) The protein expression of HIF-1a, BNIP3 and LC3B were determined with western blotting, β-actin was used as the internal control. All of the data are presented as the mean ± SEM of three independent experiments. **, P < 0.01 compared with control and #, P < 0.05 compared with H_2_O_2_.

**Additional file 1: Figure S4**


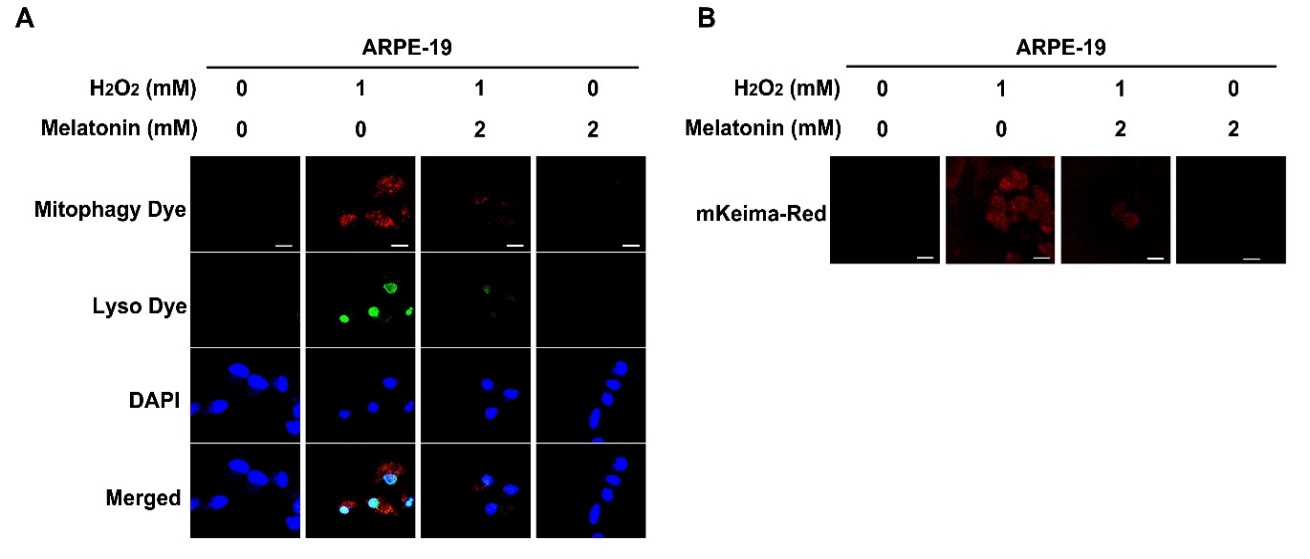


**Additional file 1: Figure S4**: (A) ARPE-19 cells were co-treated with H_2_O_2_ (1 mM) and melatonin (2 mM) for 24 h, then incubated with mitophagy dye for 15 mins by immunofluorescence assay. (B) Transfected with mitochondria-targeted fluorescent protein Keima (mt-Keima), and treated with or without melatonin (2 mM) in H_2_O_2_-treated ARPE19 cells. Representative images of Keima-Red were detected by immunofluorescence assay. Scale bars, 50 μm.
